# Healthy and Happy Working from Home? Effects of Working from Home on Employee Health and Job Satisfaction

**DOI:** 10.3390/ijerph19031122

**Published:** 2022-01-20

**Authors:** Fiona Niebuhr, Prem Borle, Franziska Börner-Zobel, Susanne Voelter-Mahlknecht

**Affiliations:** Institute of Occupational Medicine, Charité—Universitätsmedizin Berlin, Corporate Member of Freie Universität Berlin and Humboldt Universität zu Berlin, Augustenburger Platz 1, 13353 Berlin, Germany; prem.borle@charite.de (P.B.); franziska.boerner-zobel@charite.de (F.B.-Z.); susanne.voelter-mahlknecht@charite.de (S.V.-M.)

**Keywords:** COVID-19, occupational health, occupational medicine, telecommuting, telework, remote work, digitalization

## Abstract

In addition to its catastrophic health effects, the COVID-19 pandemic also acts as a catalyst for new forms of work. Working from home (WFH) has become commonplace for many people worldwide. But under what circumstances is WFH beneficial and when does it increase harms to health? The aim of this study was to investigate the influence of specific characteristics of WFH for health (work ability, stress-related physical and psychological symptoms) and job satisfaction among German employees. The study is based on data from a Germany-wide panel survey with employees from different industries (*n* = 519). Using multiple regressions, it was found that the functionality of the technical equipment at home has positive effects on the health of employees (i.e., ability to work, stress-related symptoms) and job satisfaction. The percentual weekly amount of WFH influences stress-related symptoms, i.e., a higher amount of weekly working time WFH, was associated with more stress-related symptoms. Furthermore, it negatively influences job satisfaction. The feeling of increased autonomy leads to positive effects on employees’ job satisfaction. The results provide starting points for interventions and indicate the need for legal regulations for WFH. Further theoretical and practical implications are discussed.

## 1. Introduction

Since March 2020, the increasing incidence of COVID-19 infections has led to numerous restrictions in all areas of public and private life. The working world has been confronted with multiple challenges. The coronavirus disease COVID-19, in addition to all the challenges and drastic consequences, also acts as a catalyst for new forms of work. Globally, over 3.4 billion people were restricted to their homes as of March 2020. Consequently, many millions of employees were temporarily working from home (WFH) [1]. In a European comparison, Germany lagged significantly behind other countries in regard to the possibility of WFH before the COVID-19 pandemic—less than one in two employers offered their employees the option of working from home [2]. However, COVID-19 has changed the role and conditions of home office work in Germany. For example, many companies allowed their employees to work from home to reduce the risk of infection [3]. More than half of all employees in Germany have potential access to a home office in principle, but this potential was barely realized before COVID-19 [2]. While only about 12% worked from home before the pandemic, this was possible for over 35% of Germans during the first lockdown in April 2020 [3]. The data vary modestly; [4] report that up to 26.5% of workers worked from home between March and April 2020. Hence, the proportion of employees WFH has more than doubled/almost tripled.

WFH [5] has become part of everyday life for many employees, and it can be assumed that a growing proportion of weekly working hours will continue to be performed at home in the future. In Germany, there are different regulations regarding remote workplaces. The national workplace policy only partially applies to telework, at times called telecommuting, even when a contractual agreement between employees and employer is required for these new forms of work. Hence, defining the types and conditions of WFH is crucial for regulation and research. WFH has been studied as a form of telework and mobile working or under the banner of telework, which has been defined as follows based on a comprehensive literature review: Telework is “a work practice that involves members of an organization substituting a portion of their typical work hours (ranging from a few hours per week to nearly full-time) to work away from a central workplace—typically principally from home—using technology to interact with others as needed to conduct work tasks” [6]. The number of hours may vary greatly, and policies may be defined by a minimum number of hours WFH. For instance, German policies assume location-independent working of at least ten hours per week [5]

As COVID-19 became more widespread, many governments strongly encouraged or even mandated minimizing physical presence at work [7]. EU data indicates that, in most countries, more than half of the workers who have started WFH since the pandemic had no prior experience with teleworking. Worldwide data collated by the OECD shows that countries with comparable data experienced increased rates of teleworking during the COVID-19 pandemic [8]. In France and the United Kingdom, 47% of employees teleworked during the first lockdown periods (March–May 2020). Australia also reached the same rate by December 2020. However, although similar levels of telework were reported in countries like France, the United Kingdom, and Australia with 47% of employees teleworking during the first lockdown periods, the rates of increase vary widely. In France, teleworking more than doubled compared to one year before, while it was 1.8 times the level before the pandemic in the United Kingdom and 1.5 times the level before the pandemic in Australia.

Unsurprisingly, the COVID-19 pandemic has a big impact on the views of employees and employers. In a study by Bonin and colleagues [9], up to 93% of the employees surveyed would like to have the option of WFH even after the COVID-19 pandemic. Most employees want a hybrid model with workdays in the office as well as workdays in the home office [9]. Reasons for this from the employee perspective include, e.g., increased subjectively perceived productivity, higher job satisfaction [9], higher autonomy, and better work-life balance (see e.g., [10]). From the perspective of occupational health, it is also important to assess the potential health effects of WFH. The following section summarizes empirical findings on possible health effects of WFH.

### 1.1. Employees’ Health in Home Office

Earlier studies addressed health effects of pre-pandemic telework. A systematic review by Charalampous et al. [11] found telework increased employees’ positive emotions, job satisfaction, and organizational commitment levels and ameliorated feelings of emotional exhaustion. Another systematic review suggested that telework can improve work-family life and employee well-being but that further research should explore its impact on other areas related to work life, including health and job satisfaction [12].

A growing number of studies examine the health effects of WFH during the COVID-19 pandemic. These may not be directly comparable to studies conducted before the COVID-19 pandemic that depict “normal” WFH conditions because of specific differences (e.g., rushed transformation process, low technical equipment). Studies of telework report both positive and negative health effects, and the overall effect is poorly understood, with positive health effects seemingly predominating [13].

On the one hand, telework offers some health-promoting effects. For example, the work environment at home can promote concentration, reduce the number of interruptions, and allow for more privacy [13]. WFH during COVID-19 allows some employees more flexibility in terms of working hours, which may be associated with positive psychosocial outcomes [14]. However, in another study, nearly 34% of home office workers reported that they split their work time between evenings and weekends, leading to a decrease in recovery periods [15]. This is reinforced by the general tendency for working hours to increase during WFH [9], which, in turn, can have negative health consequences, e.g., on sleep quality and physical and mental health.

An important difference between earlier telework and WFH during the COVID-19 pandemic, however, is that the switch to home office was abrupt and unplanned. As a result, some workplaces at home were set up quickly and without taking ergonomic requirements into account. Consequently, unergonomic workplaces can lead to pain or musculoskeletal diseases related to WFH (e.g., [1,16]). The most common health effects associated with telework relate to musculoskeletal problems, stress, increased work, and isolation or depression [13].

In the present study, we assess associations with stress-related symptoms and work ability, which is defined as an index that indicates how well a person can work currently and in the near future and whether that person is able to do his or her work in terms of work demands and health resources [17]. A study of employees in Australia and New Zealand found increased sedentary behavior during COVID-19 was associated with lower work ability and job performance [18]. Another study of laboratory staff in China found work ability decreased with increased job burnout during COVID-19 [19]. Moreover, a recent large-scale Finnish cohort study found that WFH was associated with decreases in self-rated health and work ability, although the effect was smaller than among employees not WFH. The same study showed that decreased work ability was associated with team reorganization due to COVID-19 [14].

WFH additionally can have a variety of other health effects. The absence of face-to-face meetings and the increase in meetings via digital platforms with the use of microphones and headphones can introduce specific strains [20]. In a study of Brazilian workers, employees who were WFH during the COVID-19 pandemic reported more vocal fatigue symptoms and muscular pain than employees who worked on-site [20]. McDowell and colleagues [21] surveyed 2303 workers in America and found that changes in work due to the COVID-19 pandemic, specifically WFH or job loss, lead to longer screen and sitting time. Such longer periods of sedentary time have negative effects on current as well as future employee health [21].

Moreover, contact restrictions to contain the COVID-19 pandemic and the resulting WFH may lead to social isolation. The majority of respondents (78%) said they missed contact with their colleagues [9]. Isolation while WFH can also have negative health effects. Isolation can lead to the feeling of loneliness, which is a stressor that is associated with various health consequences [22], such as heart disease or mental illnesses, e.g., depression and anxiety disorders [23]. Loneliness, especially prolonged loneliness with no clear end, is a major contributor to depression and suicidality [23]. In this context, loneliness can significantly reduce life satisfaction and thus increase the risk of suicide [24]. For example, in a survey of over 1000 adult Americans during the lockdown, participants reported high levels of loneliness [25]. The authors found strong associations with depression and suicidal thoughts [25].

Overall, the question of whether WFH is beneficial or detrimental to health is multi-faceted and complex. To contribute to developing practical measures in occupational medicine and to better understand the health consequences of WFH, we examine individual characteristics of WFH in this study. The focus is on the following research questions: What characteristics of working from home positively or negatively influence health, i.e., work ability and stress-related physical and psychological symptoms? And what characteristics of working from home positively or negatively influence job satisfaction?

In previous studies, survey items typically measure whether employees work from home or not. Multiple studies criticize such a dichotomous approach and call for a more precise survey of the intensity of digitalized work and WFH (see e.g., [9,26]). Therefore, in the present study, employees were asked what percentage of their current working hours they currently work from home. Bouziri and colleagues [1] conclude that, in their analysis of potential health effects from WFH during the COVID-19 pandemic, the health risks are ambivalent. On the one hand, health risks from telework could be amplified during COVID-19-related WFH because the switch to home office was very spontaneous and unplanned. Therefore, the work environment at home is probably often set up in an unergonomic way. This is compounded by a reduction in physical activity [1]. On the other hand, the duration of exposure to WFH during the COVID-19 pandemic is temporary and short-lived [1]. We hypothesize:

**Hypothesis** **1** **(H1).** *The percentage of working time in the home office (Factor 1) has a negative influence on (1a) work ability and (1b) psychological and physical stress symptoms*.

### 1.2. Employees’ Job Satisfaction in Home Office

Employees’ job satisfaction is closely associated with mental health (e.g., [27,28,29]). Job satisfaction can be defined as the degree of employees’ fulfilment with their work. Moreover, it is the positive emotional state resulting from the professional experience [27]. Temporarily working remotely can increase job satisfaction by enhancing organizational commitment and the relationship quality with leaders and decreasing work-time conflict [6]. Accordingly, the spontaneous and temporary COVID-19-related shift to WFH could have positive effects on job satisfaction. However, previous studies on the impact of WFH on job satisfaction provide ambivalent findings, such as decreased relationship quality with colleagues [6].

Social interactions at work significantly influence job satisfaction [27]. Consequently, due to the rules on social distancing and WFH during the COVID-19 pandemic, social isolation could have a negative impact on job satisfaction. In a study by Toscano and Zappalá [30], social isolation was shown to negatively impact remote job satisfaction. However, Bouziri and colleagues [1] point out that, to contain the COVID-19 pandemic, several companies switched completely to remote work and thus all employees worked from home. As a result, communication in these companies shifted entirely to digital tools. Bouziri and colleagues [1] argue that, in this case, the risk of social isolation is lower compared to what teleworkers experienced in normal times before the pandemic. The latter worked from home, while some of their colleagues had direct (social) interactions in the office.

Studies of WFH during the COVID-19 pandemic indicate ambivalent effects on employees’ health and job satisfaction. A major German public health insurance company reports that, while employees experience more autonomy and improved job satisfaction during WFH, psychological stress is increased at the same time [15]. Yet, a slight majority of mobile workers reported that their job satisfaction did not change during WFH [16]. As a possible explanation, the authors argue that the nature and extent of the job did not change during the WFH period [16]. Following these arguments, we expect only a small effect size in the influence of WFH on job satisfaction in our study and postulate:

**Hypothesis** **2** **(H2).** *The percentage of working time spent in the home office (Factor 1) has a positive influence on job satisfaction*.

### 1.3. Characteristics of Home Office

A novel focus of the present study is to investigate specific aspects of WFH. We examine four different characteristics of WFH: the technical equipment in the home office (Factor 2), the availability of a company agreement specifying the framework conditions for WFH (Factor 3), and the flexibility granted by the employer for one’s work (Factor 4) as objective characteristics of the home office. Furthermore, we focus on the experience of increased autonomy (Factor 5) as a subjective characteristic of the home office. All four characteristics are analyzed regarding their impacts on employees’ health—work ability and psychological and physical stress symptoms—and job satisfaction.

When WFH, employees are dependent on information communication technologies (ICT), which can include a wide range of technical equipment. With the onset of the COVID-19 pandemic and the spread as well as measures to contain the pandemic, many employees and companies quickly switched to WFH and other forms of mobile working at short notice. In a survey by Backhaus et al. [31], a lack of technical equipment was cited as an obstacle to WFH. In another survey, 59% of employees stated that they had no impairment in the home office due to technology [9]. Consequently, this would leave a large proportion of employees who experience impairments due to technical equipment in the home office. Yet, there is a lack of legal frameworks for how workplaces in the home office should be equipped. Since the technical equipment in the home office is not defined by law and the COVID-19-caused switch to WFH was quick and not very planned, we assume that the technical equipment in the home office is not sufficient for some employees. We therefore hypothesize that the functionality of technologies for working in home office has an influence on various health parameters as well as the job satisfaction of the employees.

**Hypothesis** **3** **(H3).** *The extent of functionality of technology (Factor 2) available in the home office has a significant positive impact on (3a) work ability, (3b) psychological and physical stress symptoms, and (3c) job satisfaction*.

Bellmann and Hübler [3] found that the job satisfaction of employees with explicit agreements for WFH was significantly higher than it was for employees without such agreements. The authors emphasize that especially times of inaccessibility should be explicitly agreed upon. Prior to the COVID-19 pandemic, significantly fewer employees worked in home offices and often contractual agreements in companies were not formulated. Due to the spontaneous and unexpected COVID-19-related move to the home office, it is reasonable to assume that many companies have not made contractual agreements regarding WFH in time [32]. In a representative survey on working hours conducted by the German Federal Institute for Occupational Safety and Health (BAuA), approximately 12% of employees stated that they had an agreement on teleworking [33]. These employees reported a greater balance between resources and burdens of work compared to employees without such agreements. For the latter, work demands such as overtime outweighed the increased flexibility as an example of workplace resources. According to [9], other important components of such a company agreement regarding WFH include documentation of working hours, agreements on setting up and financing the mobile or home workplace, data protection issues, etc. Based on these results, we hypothesize that the availability of a company agreement for WFH has a positive impact on several health parameters as well as employee job satisfaction.

**Hypothesis** **4** **(H4).** *The availability of a company agreement on working from home (Factor 3) has a positive influence on (4a) work ability, (4b) psychological and physical stress symptoms, and (4c) job satisfaction*.

WFH during the COVID-19 pandemic involves numerous strains and demands. To deal with these in a healthy way, employees need appropriate resources. We assume that flexibility in one’s own work, made possible by the employer, is an important resource for dealing with the uncertain and changing demands of work. Specifically, the participants were asked to what extent they could organize their working hours while WFH themselves, or whether arrangements with superiors were necessary. WFH can be associated with greater flexibility in terms of working hours, breaks, and sequencing of tasks, allowing a greater degree of autonomy for employees [34]. Studies indicate that a higher degree of individual responsibility, more flexibility, and freedom as central features of WFH have an effect on the job satisfaction of employees. One advantage of the increased flexibility is that employees can combine their work and private tasks and are thus freer in their work–life balance [35]. Different studies found that WFH has some health-promoting aspects, such as more flexible working hours. These can lead to higher satisfaction, performance, and motivation [4]. Therefore, we postulate:

**Hypothesis** **5** **(H5).** *The degree of flexibility that employers allow their employees while WFH (Factor 4) has a significant positive influence on (5a) work ability, (5b) psychological and physical stress symptoms, and (5c) job satisfaction.*

In addition to the resources required for WFH, this form of new work presumably also additionally entails new resources. In a meta-analysis by Gajendran and Harrison [34], telework was shown to have positive effects on perceived autonomy. The authors of [36] surveyed 709 Slovak home office workers. A significant relation between job satisfaction while teleworking and job autonomy was found. Job autonomy is linked to the feeling of having control over one’s work environment, having greater flexibility, and making a choice [37]. Petrou et al. [38] defined work autonomy as workers’ control over the performance of tasks. We assume that employees experience more autonomy in the home office than previously in the office. For example, some employees may be able to determine the order of their tasks independently of others and schedule their breaks and the start and end of work independently, etc. Wang and colleagues [39] defined work characteristics for virtual work, including social support and job autonomy. They found that “virtual work characteristics” positively influenced the performance and well-being of employees through the experienced challenges, e.g., loneliness and inefficient communication [39]. A longitudinal study also demonstrated the important role of job autonomy in times of the COVID-19 pandemic for employee health [40]. They found that emotional exhaustion among women with relatively low levels of work autonomy increased over the study period (during the COVID-19 pandemic), compared to women with high levels of work autonomy [40]. Lengen et al. [4] summarized different empirical studies that found that increased autonomy during WFH can lead to higher job satisfaction. We hypothesize the following:

**Hypothesis** **6** **(H6).** *The experience of increased autonomy (Factor 5) in the home office has a significantly positive influence on (6a) work ability, (6b) psychological and physical stress symptoms, and (6c) job satisfaction.*

## 2. Materials and Methods

Individual data from a large Germany-wide study was used to investigate the research questions. In the following, we inform about the sample, the procedure, the measurement instruments used, and their evaluation. First, descriptive analyses were performed, and correlational analyses were calculated for all variables used.

### 2.1. Procedure and Participants

The data used for the current study are part of a longitudinal study with two measurement time points. A panel company conducted a Germany-wide online survey at two measurement points (t1 July–August 2020, t2 November–December 2020). For this study, the data from the second survey were used. Using online questionnaires, employees in Germany were surveyed about, amongst other things, their current work situation and occupational health. The consent procedure and study protocol were approved by the ethics committee of Charité Universitätsmedizin Berlin. The requirement for participation was that the respondents lived in Germany, were employed, and performed a part of their weekly working time in the home office. At the time of data collection, Germany was in the so-called “lockdown light”. In this context, 14% of employees in Germany worked from home [41], i.e., approx. 6.3 million people based on 44.88 million employed persons in Germany [42]. Participants were recruited through the panel company. There was a minor financial compensation. The survey was conducted in German; sample items were translated by the authors for this article.

The data set at the second measurement point includes *n* = 519 employees. The gender distribution was rather balanced with 53.6% male and 46.4% female, and the average age was 45.37 (*SD* = 12.57), ranging from 20 to 69. The educational level of the study sample was high, a fact also shown regarding other studies of digital work based on online panels [43]: university and technical college degrees were most frequently reported as the highest professional education (39.3%). In addition, 26.8% stated that they had completed dual vocational training or skilled worker training. A total of 18.7% indicated a degree from an undergraduate technical college, 2.7% no vocational training degree, and 2.3% a doctorate or postdoctoral degree. The average monthly net income (i.e., the sum of wages, salary, income, in each case after deduction of taxes etc.) was 2000–3000€. The sample was also diverse in terms of reported branches. The following information was provided regarding branches (in descending order): 12.7% other services; 10.4% other industries; 9.4% IT, computers, and mathematics; 8.3% social work and education; 8.1% office, business, and administration; 7.1% banking, insurance, and real estate; 6.4% health, medicine, nursing, and sports; 5.6% trade, distribution, sales; and all other industries <5%. Participants also provided information on their employment. The majority of the sample (68.2%) was employed on a permanent basis.

### 2.2. Measures

#### 2.2.1. Factors of Working from Home

The amount of worktime in the home office was measured by a single item, which was developed by the research team. Participants were asked to indicate on a sliding scale of 0–100% what percentage of their weekly work time they currently work from home. Furthermore, the technical functionality of applications used in the home office (“the technical applications I need for my work function”) and the experience of having more autonomy in the home office (“I experience more autonomy/decision-making in the home office”) were measured each with a single-item using a five-point Likert scale ranging from 1 (does not apply at all) to 5 (fully applies). The existence of an agreement regarding WFH was measured with one item. The response options included additional information about the timing of the agreement: 1 (no agreement), 2 (yes, even before the COVID-19 pandemic), or 3 (yes, only since the COVID-19 pandemic). Finally, participants were asked, if and to what degree of flexibility their employers allow them to WFH. The response options were “No, I am not allowed to work in a home office”, “Yes, with clear guidelines”, “Yes, flexible with agreement”, and “Yes, completely flexible to schedule myself”; i.e., higher values indicate a higher degree of flexibility in the home office that is granted by employers.

#### 2.2.2. Outcome Variables

The general level of work ability was assessed using one item from the Work Ability Index (WAI, [44]). Ebener and Hasselhorn [17] point out that the use of this single item as the Work Ability Score (WAS) is the best known in occupational health research. Therefore, general work ability was assessed on a sliding scale from 0 (totally unable to work) to 10 (best work ability). Using items from the subscale “psychological and physical symptoms of stress” of the Burnout Bullying Inventory [45], 11 different physical and psychological symptoms (e.g., headaches, listlessness, sadness, and nightmares) were assessed on a five-point Likert scale ranging from 1 (does not apply at all) to 5 (fully applies). For hypothesis testing, a sum score was formed across all symptoms (range: 11–55), with higher scores representing stronger agreement with symptoms and, consequently, a greater variety of symptoms. Job satisfaction was measured by a single item (“I am satisfied with my job.”) using a five-point Likert scale ranging from 1 (does not apply at all) to 5 (fully applies).

#### 2.2.3. Statistical Analyses

All analyses were conducted with the software IBM SPSS Statistics 26.0 (International Business Machines Corporation, Armonk, NY, USA). Prior to testing the hypotheses, descriptive analyses were conducted. The correlations of all outcome variables and predictors are shown in Table 1. A multiple regression model was performed to test the influence of the pre-described factors on the outcome variables, i.e., work ability, stress-related symptoms, and job satisfaction. 

## 3. Results

The mean values and standard deviations of the used items are shown in Table 1. For hypothesis testing, a sum score of all psychological and physical symptoms was formed. The estimate of reliability (internal consistency) for the subscale to assess stress-related symptoms is α = 0.918.

### 3.1. Testing the Hypotheses

#### Multiple Regression Models

Due to the complexity of health, we examined multiple regression analyses models. Specifically, we examined the influence of the WFH-characteristics on various outcome variables, i.e., work ability, stress-related symptoms, and job satisfaction. The results of the multiple regression models are shown in Table 2. First, we tested the influence of multiple predictors on employees’ work ability. Factor 2 (technical functionality) was the only significant predictor of work ability and explained 8.2% of the variance in employees’ work ability, *F*(5, 513) = 10.296, *p* < 0.001. Therefore, Hypothesis 3a was confirmed. Second, we tested the influence of the predictors on different physical and psychological stress-related symptoms. The predictors Factor 1 (percentage of working time spent in the home office), Factor 2 (technical functionality), and Factor 3 (availability of company agreement) significantly predicted stress-related symptoms, *F*(5, 513) = 4.483, *p* < 0.01, corrected *R*^2^ = 0.033. Surprisingly, a positive beta weight for Factor 3 was shown, i.e., employees with a higher level of contractual arrangements to WFH reported more stress-related symptoms. Therefore, Hypotheses 1b and 3b were confirmed, whereas the analysis for Hypothesis 4b showed an opposite effect. Finally, we analyzed job satisfaction in a multiple regression. Factor 1, Factor 2, and Factor 5 (experience of increased autonomy) significantly predicted job satisfaction, *F*(5, 513) = 10.08, *p* < 0.001, *R*^2^ = 0.081. Accordingly, Hypotheses 2, 3c, and 6c were confirmed. The results of the multiple regressions are presented in Table 2.

## 4. Discussion

The COVID-19 pandemic is having a far-reaching impact on lives worldwide. The consequences for the world of work are drastic, and, at the same time, the pandemic is serving as a catalyst for new forms of work and accelerating digitalization. The aim of this study was to increase knowledge about the circumstances under which working from home (WFH) is beneficial to employees’ health and job satisfaction. Considering different and partly contradictory empirical findings on the question of whether WFH is beneficial or detrimental to health, we examined specific factors of WFH and their health effects and influences on job satisfaction. In a Germany-wide online survey, we investigated the effects of five WFH characteristics (i.e., the percentage of weekly working time WFH, the functionality of the technology, the existence of a company regulation, the degree of flexibility enabled by the employer, and the experience of autonomy) on health parameters (i.e., work ability and stress-related psychological and physical symptoms) as well as on employees’ job satisfaction. Following Bonin et al. [9], we examined the intensity of home office work (i.e., the extent of percent time worked in the home office), instead of dichotomously modeling WFH.

The percentage of weekly working time WFH had a negative influence on stress-related symptoms in the present study. Accordingly, employees who work a higher percentage of their weekly hours from home reported more psychological and physical symptoms than employees who WFH fewer hours. In contrast, the percentage of weekly work time spent in the home office had no influence on work ability (Hypothesis 1a). Consistent with the findings of Moretti et al. [16] and contrary to our reasoning, the amount of WFH had a negative impact on employee job satisfaction (Hypothesis 2). It is often believed that telework enhances job satisfaction, yet research has found both positive and negative relationships. Golden and Vega [46], however, have suggested that job satisfaction may plateau and decrease at more extensive levels of telework. The extent of functionality of technology positively predicted work ability (Hypothesis 3a), physical and psychological stress-related symptoms (Hypothesis 3b), and job satisfaction (Hypothesis 3c). The important influence of this factor on all outcome variables reinforces previous findings in an EU-wide study highlighting the importance of an adequate provision of equipment. The same study also found that, among employees with good equipment, 77% were satisfied with telework compared to 31% of those without the appropriate equipment [47]. Overall, 39% of respondents reported having regulations on WFH in their employment contract. Contrary to our expectations, the availability of a company agreement on WFH showed associations with a higher number of stress-related symptoms. This may be due to the fact that the variable used did not assess the quality of agreements, although telecommuting policies and procedures vary across organizations in terms of allowable practices and specificity and thus entail varied outcomes [6].

Subjectively perceived increases in autonomy while WFH are important for employees’ health during the COVID-19 pandemic, as shown by Meyer and colleagues [40]. Contrary to previous findings [40] the experience of increased autonomy in the home office did not affect work ability (Hypothesis 6a) or stress-related symptoms (Hypothesis 6b) in our study. As postulated, however, the experience of increased autonomy positively predicted job satisfaction (Hypothesis 6c), which is consistent with findings in the meta-analysis by Gajendran and Harrison [34]

### 4.1. Implications

Even after the COVID-19 pandemic or the lifting of COVID-19-related restrictions, WFH is likely to remain an essential part of the new world of work. Due to the experienced benefits of WFH, both by employers and employees, and technological advances, the trend toward WFH could continue beyond the end of the pandemic (e.g., [48]). In the future, many employees may want a hybrid work model—a flexible mix of WFH and on-site work (e.g., [2]). In preparation for this possible development, the health effects of telework and hybrid work should be studied in more detail to identify potential risk factors and develop appropriate workplace policies [6].

The functionality of the technical equipment in the home office has a decisive influence on the health and job satisfaction of employees. Due to the mostly unplanned transformation process towards WFH, the technical equipment at many people’s homes was not sufficient to enable them to work effectively and healthily. The work situation could still improve as both companies and individuals improve their technical equipment for WFH. In our study, the factor “functionality of technical equipment” influenced all outcomes. In order to protect and strengthen the health and work experience of employees during WFH, legal regulations should be developed. Certain regulations, such as insurance coverage in the event of accidents, also apply to WFH. Nevertheless, there is a need for coordination for more concrete regulations, not only in Germany. Analogous to regulations regarding telework, employers should provide the necessary technical equipment and ensure its functionality. It would be conceivable, for example, that employers provide mobile, ergonomically designed work equipment for WFH in the interest of occupational health and safety.

The findings of this study, as well as previous studies, should be used to develop practical measurement tools for occupational health. Specific factors of WFH as well as anticipated consequences thereof should be considered. Measuring WFH as a percentage of weekly working hours also allows hybrid work to be modeled.

The subjectively perceived increase in one’s own autonomy was surveyed as a job resource. WFH gives employees more autonomy, which is needed to cope with the demands of this new form of work. The present study showed the importance of autonomy for job satisfaction. In future crises, the autonomy of employees should be increased as quickly as possible from the beginning [40]. Flexible work arrangements that enable WFH and hybrid work models, as well as a certain degree of technical flexibility, can empower employees in crises and possibly protect their health. Autonomy in crises gives employees the opportunity to better manage private and professional demands [40]. Future studies should therefore examine the function of job-related resources as well as personal resources in the context of new forms of work, especially WFH. Companies should promote relevant resources (e.g., social support and feedback) that are necessary for working healthily and satisfied in times of crisis as well as in the context of new forms of work.

### 4.2. Limitations

As with any field study, this study has some limitations. First, the data used here are from a single source and are self-reported. This can lead to monomethod bias and carries the risk of social desirability bias and conceptual overlap to the extent that self-reported measures express a similar underlying meaning as the outcome measures (see e.g., [49]).

Second, causal interpretations cannot be made based on our cross-sectional study design. The study conducted two surveys with the same sample but did not use completely identical questionnaires. Therefore, the analysis for this article was cross-sectional with data from one measurement point. Longitudinal study designs with multiple measurement time points would be necessary to make causal statements. An analysis of the data from both the first and second measurement time point by the research team is still pending. In addition, there is a lack of baseline data regarding employee health, job satisfaction, etc., prior to the onset of the COVID-19 pandemic because the data were collected during the lockdown in the winter of 2020.

Third, most factors and outcome variables were tested using a single item. We chose this approach for economic reasons. However, this questionnaire design significantly reduces the variance measured. In addition, no latent factors could be formed since relevant factors were represented by only one item each. In future studies, specific factors of WFH should be selected and surveyed using subscales. Furthermore, we used some items that have not yet been validated. The use of established (sub)scales would certainly be beneficial in future studies but was hardly possible due to the topicality of the COVID-19 situation, the novelty, and the exploratory nature of the study at baseline.

Furthermore, the study was conducted exclusively in Germany. To confirm the results, future studies should also survey employees in other countries. To ensure a certain generalizability, employees from different industries were surveyed, thus reflecting a certain diversity of the German working population.

Finally, research questions about WFH were examined in the context of the COVID-19 pandemic, an anxiety-producing, uncertain situation. Interpretation of study findings on the health effects of WFH conducted during the pandemic should take this into account. WFH-associated health risks could be amplified by the context of the COVID-19 pandemic [1]. The influence of concern about the virus may play a critical role in the current association of WFH and health outcomes as well as job satisfaction. For example, [30] found that social isolation had more negative effects on job satisfaction among employees with high levels of virus-related concern (i.e., the feeling of being worried, frightened, depressed, or angry) than among employees with lower levels of virus-related anxiety.

## 5. Conclusions

The COVID-19 pandemic led to rapid changes worldwide and had an enormous impact on the world of work. The uptake of WFH was accelerated by the COVID-19 pandemic and has increased to unprecedented levels. Characteristic factors of WFH, such as the functionality of technology, the percentage of working time WFH, or the experience of autonomy are associated with health and job satisfaction. Sufficient and functioning technical equipment as well as a high degree of autonomy are important for healthy and satisfied work from home. Both should be ensured by appropriate legal regulations. Work climate, social support, and (informal) exchange should be considered while WFH collectively. Many employees wish to continue working from home (at least partially) even after the pandemic has ended. Understanding the factors of WFH and their influences on the job satisfaction and health of employees allows targeted interventions and legal regulations to be developed and implemented. With this knowledge, employers and employees can take appropriate measures to design digital work in a healthy and satisfying way.

## Figures and Tables

**Table 1:** Means (M), standard deviations (SD) and correlations (r) for the Main Variables.

	Variable	*M*	*SD*	1	2	3	4	5	6	7
1	Percentage of working time WFH	57.62	40.71							
2	Work ability	8.69	1.89	0.112 *						
3	Symptoms	21.94	7.94	0.026	−0.490 **					
4	Job satisfaction	3.74	1.08	0.098 *	0.442 **	−0.355 **				
5	Agreement	1.68	0.87	0.145 **	−0.024 *	0.115 **	−0.035			
6	Flexibility	2.70	1.14	0.614 **	0.159 **	−0.074	0.170 **	0.031		
7	Functionality of technical equipment	3.84	1.31	0.541 **	0.294 **	−0.114 **	0.265 **	0.042	0.533 **	
8	Increased autonomy	3.36	1.37	0.561 **	0.174 **	−0.018	0.239 **	0.088 *	0.548 **	0.648 **

Note. *n* = 519. * *p* < 0.05. ** *p* < 0.01.

**Table 2:** Results of multiple regression analysis.

Dependent Variable	Predictors	Coefficient (SE)	Standardized Coefficient	*R*^2^ (Full Model)
Work ability	Factor 1 ^a^	−0.004 (0.003)	−0.077	0.082
	Factor 2 ^b^	0.464 *** (0.084)	0.322	
	Factor 3 ^c^	−0.055 (0.091)	−0.026	
	Factor 4 ^d^	0.07 (0.094)	0.043	
	Factor 5 ^e^	−0.017 (0.083)	−0.012	
Stress-related symptoms	Factor 1 ^a^	0.025 * (0.012)	0.131	0.033
	Factor 2 ^b^	−1.109 ** (0.365)	−0.183	
	Factor 3 ^c^	0.902 * (0.393)	0.101	
	Factor 4 ^d^	−0.689 (0.407)	−0.099	
	Factor 5 ^e^	0.420 (0.357)	0.072	
Job satisfaction	Factor 1 ^a^	−0.003 * (0.002)	−0.127	0.081
	Factor 2 ^b^	0.175 (0.049)^***^	0.211	
	Factor 3 ^c^	−0.049 (0.052)	−0.040	
	Factor 4 ^d^	0.054 (0.054)	0.057	
	Factor 5 ^e^	0.116 * (0.047)	0.146	

Note. *n* = 519; * *p* < 0.05. ** *p* < 0.01. *** *p* < 0.001; ^a^ Factor 1: percentage of working time spent in the home office; ^b^ Factor 2: extent of functionality of technology available in the home office; ^c^ Factor 3: availability of a company agreement on WFH; ^d^ Factor 4: degree of flexibility in home office granted by the employer; ^e^ Factor 5: experience of increased autonomy in the home office.

## Data Availability

The data presented in this study are available on reasonable request from the corresponding author, F.N. The data are not publicly available, as this was assured to the participants in the study information as well as in the data privacy statement.

## References

[1] Bouziri H., Smith D.R., Descatha A., Dab W., Jean K. (2020). Working from home in the time of COVID-19: How to best preserve occupational health?. Occup. Environ. Med..

[2] Alipour J., Falck O., Schüller S. (2020). Homeoffice während der Pandemie und die Implikationen für eine Zeit nach der Krise. Ifo Schnelld..

[3] Homeoffice Braucht Klare Regeln. https://www.iab-forum.de/homeoffice-braucht-klare-regeln/.

[4] Lengen J.C., Kordsmeyer A.C., Rohwer E., Harth V., Mache S. (2021). Soziale Isolation im Homeoffice im Kontext der COVID-19-Pandemie. Zent. Arb. Arb. Ergon..

[5] Kniffin K.M., Narayanan J., Anseel F., Antonakis J., Ashford S.P., Bakker A.B., Bamberger P., Bapuji H., Bhave D.P., Choi V.K. (2021). COVID-19 and the workplace: Implications, issues, and insights for future research and action. Am. Psychol..

[6] Allen T.D., Golden T.D., Shockley K.M. (2015). How effective is telecommuting? Assessing the status of our scientific findings. Psychol. Sci. Public Interest.

[7] Milasi S., Fernandez-Macias E., Gonzalez-Vasques I. (2020). Telework in the EU before and after the COVID-19: Where we were, where we head to. Eur. Union.

[8] OCED (2021). Teleworking in the COVID-19 Pandemic: Trends and Prospects.

[9] Bonin H., Eichhorst W., Kaczynska J., Kümmerling A., Rinne U., Scholten A., Steffens S. (2020). Kurzexpertise: Verbreitung und Auswirkungen von Mobiler Arbeit und Homeoffice: Erstellt im Auftrag des Bundesministeriums für Arbeit und Soziales.

[10] Beermann B., Backhaus N., Hünefeld L., Janda V., Schmitt-Howe B., Sommer S. (2020). Veränderungen in der Arbeitswelt–Reflexion des Arbeitsschutzsystems.

[11] Charalampous M., Grant C.A., Tramontano C., Michailidis E. (2019). Systematically reviewing remote e-workers’ well-being at work: A multidimensional approach. Eur. J. Work Organ. Psychol..

[12] de Macêdo T.A.M., Cabral E.L.D.S., Silva Castro W.R., de Souza Junior C.C., da Costa Junior J.F., Pedrosa F.M., da Silva A.B., de Medeiros V.R.F., de Souza R.P., Cabral M.A.L. (2020). Ergonomics and telework: A systematic review. Work.

[13] Tavares A.I. (2017). Telework and health effects review. Int. J. Healthc..

[14] Ervasti J., Aalto V., Pentti J., Oksanen T., Kivimäki M., Vahtera J. (2021). Association of changes in work due to COVID-19 pandemic with psychosocial work environment and employee health: A cohort study of 24 299 Finnish public sector employees. Occup. Environ. Med..

[15] Göpner-Reinecke C. (2019). Arbeiten im Homeoffice-Höhere Arbeitszufriedenheit, aber Stärkere Psychische Belastung. Wissenschaftliches Institut der AOK (WIdO) und AOK-Bundesverband Pressemitteilung zum Fehlzeiten-Report 2019.

[16] Moretti A., Menna F., Aulicino M., Paoletta M., Liguori S., Iolascon G. (2020). Characterization of home working population during COVID-19 emergency: A cross-sectional analysis. Int. J. Environ. Res. Public Health.

[17] Ebener M., Hasselhorn H.M. (2019). Validation of Short Measures of Work Ability for Research and Employee Surveys. Int. J. Environ. Res. Public Health.

[18] Hunter J.R., Meiring R.M., Cripps A., Suppiah H.T., Vicendese D., Kingsley M.I., Gordon B.A. (2021). Relationships between Physical Activity, Work Ability, Absenteeism and Presenteeism in Australian and New Zealand Adults during COVID-19. Int. J. Environ. Res. Public Health.

[19] Lu Y., Liu Q., Yan H., Gao S., Liu T. (2021). Job burnout and its impact on work ability in biosafety laboratory staff during the COVID-19 epidemic in Xinjiang. BMC Psychiatry.

[20] Siqueira L.T.D., Dos Santos A.P., Silva RL F., Moreira PA M., da Silva Vitor J., Ribeiro V.V. (2020). Vocal self-perception of home office workers during the COVID-19 pandemic. J. Voice.

[21] McDowell C.P., Herring M.P., Lansing J., Brower C., Meyer J.D. (2020). Working from home and job loss due to the COVID-19 pandemic are associated with greater time in sedentary behaviors. Front. Public Health.

[22] Hawkley L.C., Cacioppo J.T. (2010). Loneliness matters: A theoretical and empirical review of consequences and mechanisms. Ann. Behav. Med..

[23] Stickley A., Koyanagi A. (2016). Loneliness, common mental disorders and suicidal behavior: Findings from a general population survey. J. Affect. Disord..

[24] Koivumaa-Honkanen H., Honkanen R., Viinamaeki H., Heikkilae K., Kaprio J., Koskenvuo M. (2001). Life satisfaction and suicide: A 20-year follow-up study. Am. J. Psychiatry.

[25] Killgore W.D., Cloonan S.A., Taylor E.C., Dailey N.S. (2020). Loneliness: A signature mental health concern in the era of COVID-19. Psychiatry Res..

[26] Borle P., Boerner-Zobel F., Voelter-Mahlknecht S., Hasselhorn H.M., Ebener M. (2021). The social and health implications of digital work intensification. Associations between exposure to information and communication technologies, health and work ability in different socio-economic strata. Int. Arch. Occup. Environ. Health.

[27] Bulińska-Stangrecka H., Bagieńska A. (2021). The role of employee relations in shaping job satisfaction as an element promoting positive mental health at work in the era of COVID-19. Int. J. Environ. Res. Public Health.

[28] Hakanen J.J., Peeters M.C., Schaufeli W.B. (2018). Different types of employee well-being across time and their relationships with job crafting. J. Occup. Health Psychol..

[29] Wright T.A., Cropanzano R., Bonett D.G. (2007). The moderating role of employee positive well being on the relation between job satisfaction and job performance. J. Occup. Health Psychol..

[30] Toscano F., Zappalà S. (2020). Social isolation and stress as predictors of productivity perception and remote work satisfaction during the COVID-19 pandemic: The role of concern about the virus in a moderated double mediation. Sustainability.

[31] Backhaus N., Tisch A., Kagerl C., Pohlan L. (2020). Arbeit von zuhause in der Corona-Krise: Wie geht es weiter?.

[32] Mojtahedzadeh N., Rohwer E., Lengen J., Harth V., Mache S. (2021). Gesundheitsfördernde Arbeitsgestaltung im Homeoffice im Kontext der COVID-19-Pandemie. Zbl Arbeitsmed..

[33] BAuA SARS-CoV-2-Arbeitsschutzregel. https://www.baua.de/DE/Angebote/Rechtstexte-und-Technische-Regeln/Regelwerk/AR-CoV-2/AR-CoV-2.html.

[34] Gajendran R.S., Harrison D.A. (2007). The good, the bad, and the unknown about telecommuting: Meta-analysis of psychological mediators and individual consequences. J. Appl. Psychol..

[35] Kröll C., Nüesch S. (2019). The effects of flexible work practices on employee attitudes: Evidence from a large-scale panel study in Germany. Int. J. Hum. Resour. Manag..

[36] Karácsony P. (2021). Impact of teleworking on job satisfaction among Slovakian employees in the era of COVID-19. Probl. Perspect. Manag..

[37] Slemp G.R., Kern M.L., Vella-Brodrick D.A. (2015). Workplace Well-Being: The Role of Job Crafting and Autonomy Support. Psychol. Well-Being.

[38] Petrou P., Demerouti E., Peeters M.C., Schaufeli W.B., Hetland J. (2012). Crafting a job on a daily basis: Contextual correlates and the link to work engagement. J. Organ. Behav..

[39] Wang B., Liu Y., Qian J., Parker S.K. (2021). Achieving effective remote working during the COVID-19 pandemic: A work design perspective. Appl. Psychol..

[40] Meyer B., Zill A., Dilba D., Gerlach R., Schumann S. (2021). Employee psychological well-being during the COVID-19 pandemic in Germany: A longitudinal study of demands, resources, and exhaustion. Int. J. Psychol..

[41] Statista Anteil der im Homeoffice Arbeitenden Beschäftigten in Deutschland vor und Während der Corona-Pandemie 2020 und 2021. https://de.statista.com/statistik/daten/studie/1204173/umfrage/befragung-zur-homeoffice-nutzung-in-der-corona-pandemie/.

[42] Statistisches Bundesamt Erwerbstätigkeit 2020: Aufwärtstrend am Arbeitsmarkt Nach 14 Jahren Beendet. https://www.destatis.de/DE/Presse/Pressemitteilungen/2021/01/PD21_001_13321.html.

[43] Borle P., Reichel K., Voelter-Mahlknecht S. (2021). Is There a Sampling Bias in Research on Work-Related Technostress? A Systematic Review of Occupational Exposure to Technostress and the Role of Socioeconomic Position. Int. J. Environ. Res. Public Health.

[44] Hasselhorn H.M., Freude G., Wirtschaftsverl N.W. (2007). Der Work-Ability-Index: Ein Leitfaden.

[45] Satow L. (2013). BMI-Burnout-Mobbing-Inventar. https://www.psycharchives.org/en/item/404c5b05-72cf-4646-93c9-b83a5634dd98.

[46] Golden T.D., Veiga J.F. (2005). The Impact of Extent of Telecommuting on Job Satisfaction: Resolving Inconsistent Findings. J. Manag..

[47] Predotova K., Vargas Llave O. (2021). Working Conditions and Sustainable Work. Workers Want to Telework but Long Working Hours, Isolation and Inadequate Equipment Must Be Tackled.

[48] Mattioli D., Putzier K. (2020). Someday the coronavirus pandemic will release its grip on our lives and we will return to the workplace. The question is: Will there be an office to go back to when this is all over?. Wall Str. J..

[49] Borle P., Reichel K., Niebuhr F., Voelter-Mahlknecht S. (2021). How Are Techno-Stressors Associated with Mental Health and Work Outcomes? A Systematic Review of Occupational Exposure to Information and Communication Technologies within the Technostress Model. Int. J. Environ. Res. Public Health.

